# Radiofrequency ablation* vs.* stereotactic body radiotherapy for stage IA non-small cell lung cancer in nonsurgical patients

**DOI:** 10.7150/jca.51413

**Published:** 2021-03-19

**Authors:** Ming Li, Xiao Xu, Yingyi Qin, Peng Zhang, Changxing Shen, Qing Xia, Lihong Fan

**Affiliations:** 1 Department of Respiratory Medicine, Shanghai 10th People's Hospital, Tongji University School of Medicine, Shanghai 200072, China.; 2 Shanghai Clinical College, Anhui Medical University, Hefei 230032, Anhui Province, China.; 3 Department of Health Statistics, Second Military Medical University, Shanghai 200433, China.; 4 Department of Cardio-Thoracic Surgery, Shanghai 10th People's Hospital, Tongji University School of Medicine, Shanghai 200072, China.

**Keywords:** radiofrequency ablation, stereotactic body radiotherapy, non-small-cell lung carcinoma, survival

## Abstract

**Background:** Approximately 20% resectable non-small cell lung cancer (NSCLC) patients are treated non-surgically due to various reasons. The aim of the present study was to compare the effectiveness of radiofrequency ablation (RFA) and stereotactic body radiotherapy (SBRT) in patients with stage IA NSCLC who were ineligible for surgery using the surveillance, epidemiology and end-results (SEER) Database.

**Methods:** Using the SEER registry, we identified a total of 6,195 IA NSCLC patients who received SBRT or RFA between 2004 and 2015 because of ineligibility for surgical resection due to various reasons. Complete clinical information was available in all these patients. Overall survival (OS) and cancer-specific survival (CSS) were compared between RFA and SBRT groups by using propensity score matching (PSM), inverse probability of treatment weight (IPTW), and overlap weighting analysis. Additionally, an exploratory analysis was conducted to determine the effectiveness of RFA treatment based on the subsets of clinically relevant patients.

**Results:** Of the 6,195 nonsurgical IA NSCLC patients, 191 patients (3.1%) received RFA and the other 6,004 patients (96.9%) received SBRT. The one-, three- and five-year OS in the unmatched RFA and SBRT groups were 83.3%, 48.5%and 29.1%* vs.* 83.8%, 48.3% and 27.4%, respectively, there was similar results in the PSM, IPTW, overlap weighing analysis. Nonsurgical IA NSCLC patients receiving RFA seemed to have better five-year survival than those receiving SBRT, though the difference was not statistically significant (OS, HR; 0.986; 95% CI, 0.827-1.175, *P*=0.8738; CSS, HR; 0.965; 95% CI, 0.765-1.219, *P*=0.7663). We found that the odds of receiving RFA decreased with larger tumor size (>2, <3 cm, OR; 0.303; 95% CI, 0.191-0.479; >3 cm, OR; 0.153; 95% CI, 0.093-0.251) compared with tumor size <1 cm. In subgroup analysis, patients receiving RFA seemed to have better OS than those receiving SBRT, though the difference was not statistically significant. This specific trend was even more obvious in patients with tumors <1cm in diameter (*P*=0.1577).

**Conclusion:** In comparison with SBRT, RFA did not seem to adversely affect CSS and OS of IA NSCLC patients who were not suitable for surgical treatment. In addition, RFA seemed to offer better survival to IA NSCLC patients, especially those with tumors <1 cm.

## Introduction

According to cancer statistics 1, there will be 606,880 patients' whose lives will be claimed by cancers in the USA in 2019, suggesting that about 1,700 cancer-related deaths will occur every day, among which lung cancer is a major death-inducing factor causing about 25% of all cancer-related deaths. To about 16% early diagnosed non-small cell lung cancer (NSCLC) patients, lobectomy resection remains the first-line treatment 2. However, due to continuously increasing comorbidities or weakened cardiopulmonary functions, even sublobar resection is not suitable for some old or other high-risk patients, let alone a pulmonary resection 3. Based on the results of current treatment, early diagnosed inoperable lung cancer patients generally have an unsatisfactory performance in the primary tumor control rate ranging between 30% and 40%, and present a high mortality rate, with a three-year overall survival (OS) rate between 20% and 35%. Stereotactic body radiotherapy (SBRT) is a common conventional non-surgical option for those early diagnosed NSCLC patients who are considered inoperable. Nonetheless, the outcome has been shown unsatisfactory, only with a five-year OS rate about 12% and a mean survival duration about 20 months 4,5.

Radiofrequency ablation (RFA) is the latest and promising treatment for cancer patients, including inoperable NSCLC patients 6. With high feasibility and safety, RFA is able to cause irreversible injury or coagulation necrosis to tumors by utilizing the heat biological effect 7. Recently, more hospitals are choosing RFA rather SBRT as a non-surgical treatment option for early diagnosed NSCLC 8. Presently, there are few data to compare the outcomes of these two non-surgical treatment approaches. The aim of the present study was to compare the effectiveness of SBRT and RFA in stage IA NSCLC patients who are deemed ineligible for surgery by using the SEER Database.

## Methods

### Data source

Using the National Cancer Institute SEER database of (http://seer.cancer.gov/data/options.html), a retrospective analysis was conducted by exempting the data from the institutional review board's oversight. The project of SEER was initiated in 1973 in the USA as population-based registry of cancer which involves about one-tenth of the population in the country. The patient sample of the presented study was selected from the de-identified patients in the NCI SEER 18 Registries (SEER*Stat Database: Incidence-SEER. 18 Custom Data [with additional treatment fields] Nov 2016 Sub), whose data included no personal identifiers and were submitted to the NCI through electronic channels, which are allowed to be used in relevant medical research. The researchers of the present study had obtained the approval from the ethics committee and the institutional review board before using these de-identified data, including the clinicopathological features of the patients, tumor histology, cancer stage, timing and type of the first course treatment, and the therapeutic outcomes. A yearly follow-up rate of 90% for all involved patients whose cancer was diagnosed in recent five years was required for accreditation.

### Study population

**Figure 1:**
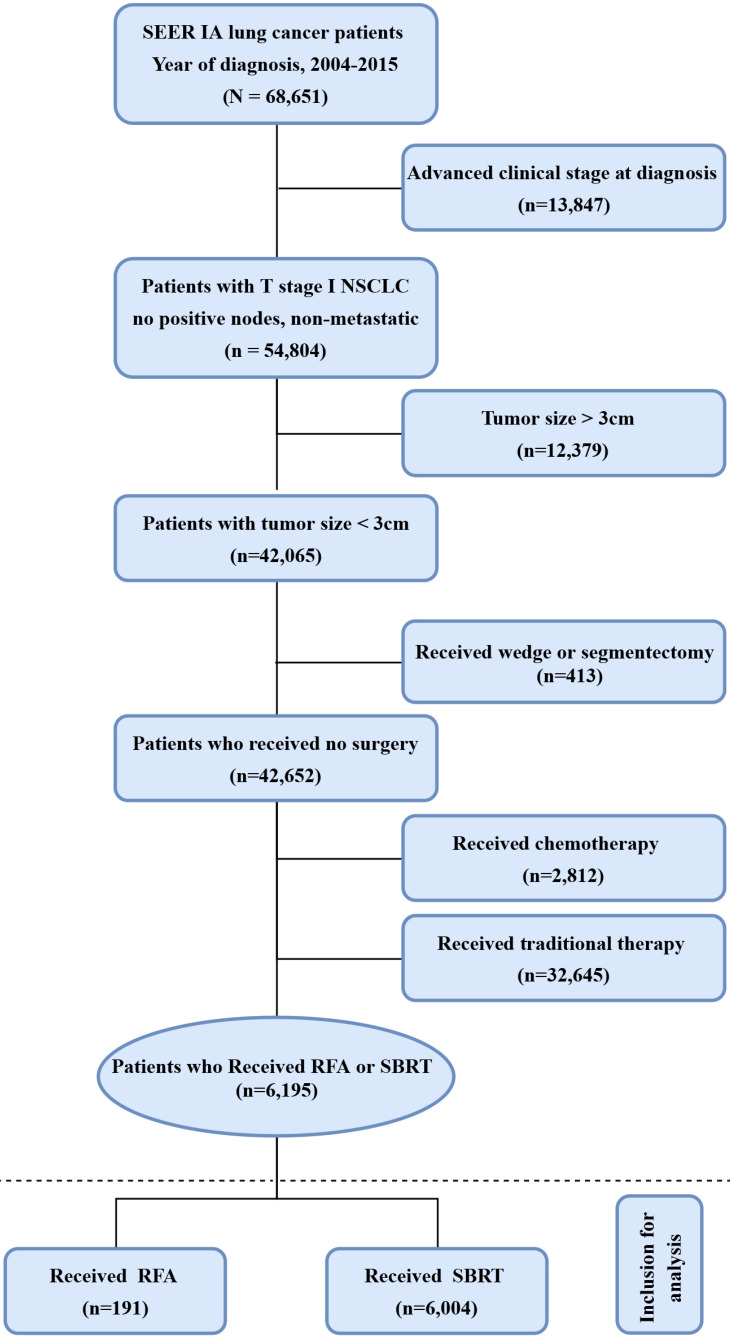
Flow chart of patient selection for the study. Abbreviations: RFA: radiofrequency ablation; SBRT: stereotactic body radiotherapy.

Inclusion: Of the 618,830 male and female patients with primary NSCLC who were diagnosed with lung adenocarcinoma (AD) (histological codes 8244, 8245, 8250-8255, 8260, 8290, 8310, 8323, 8333, 8480, 8481, 8490, 8507, 8550, 8570, 8571, 8574, and 8576), squamous cell carcinoma (SQCC) (histologic codes 8052, 8070-8075, 8083, 8084, 8123), and large cell carcinoma (LCC) histological codes 8012-8014, 8046, 8050, 8003, 8004, 8022, 8031-8035, 8082, 8200, 8240, 8249, 8430, 8560, 8562, 8980) during the 10-year period from 2004 to 2015, 68,262 patients with clinical stage I NSCLC (T1N0M0) who had not received any surgical treatment such as hepatectomy, surgical resection, extended lobectomy and lobectomy but received either SBRT or RFA were selected as the subjects of the present study. Exclusion: Patients who had been treated with (neo-)adjuvant chemotherapy or had unknown chemotherapy information were excluded from the study. Finally, 6,195 patients who met the above inclusion and exclusion criteria were recruited for the present study (Figure 1).

### Covariates

During the analysis, variables on the tumor, facility and patient level were considered. Variables on the tumor level included the year of diagnosis (2004-2007, 2008-2011 and 2012-2015), tumor size and TNM stage, with the tumor grade available to patients undergoing biopsy for tumor evaluation. Variables on the facility level included the geographic region, quarterly case volume, distance from the treatment facilities to the residence of the patients, and facility type as assigned by the Cost Optimization Committee (COC). Major treatment facilities could be seen in the accreditation category assigned by the COC on the basis of services available and the case volume, including the integrated network cancer program, comprehensive community and community. Variables on the patient level included the race, gender, age at the time of diagnosis, the status of insurance, percentages of patients with the highest education under high school, and the median family income based on the patient ZIP code. The SEER data dictionary has provided a comprehensive description of all included variables for reference.

### Objectives

The present study was conducted primarily to compare OS and cancer-specific survival (CSS) in early diagnosed inoperable NSCLC patients who underwent the treatment of SBRT or RFA. The CSS and OS were defined as the period from the date of diagnosis to the date of death of the patients. Besides, this study also tried to determine the effectiveness of RFA treatment based on the subsets of clinically relevant patient.

### Statistical analysis

Wilcoxon Rank Sum test, Chi-square test, or Cochran-Mantel-Haenszel (CMH) test was applied to assess the correlations between different treatment methods and all the variables mentioned above. The primary outcomes included the survival rate and survival curve estimated on the basis of the Kaplan-Meier estimator. OS and CSS between SBRT and RFA were compared by using univariable and multivariable Cox proportional hazards models adjusted for all the baseline covariates. Additionally, three PS models (PSM, IPTW and overlap weighting method) were used in our study. The PS of receiving RFA was estimated via a multivariable logistic regression model. The final PS model was determined by using stepwise variable selection on the condition of *P*<0.20 to be initially included, and *P*>0.10 to eventually remain in the model. Patients in the SBRT and RFA groups were matched based on the nearest available matching method with a ratio of 3:1. The calculation of a stabilized inverse probability of treatment weight was performed based on the score of propensity. IPTWs were truncated at the 5^th^ and 95^th^ percentiles. The overlap weights were constructed according to method suggested by Fan Li et al 8. The weights for SBRT were PSRFA, and the weights for SBRT were (1-PSRFA). For the PS models of OS and CSS, all patients were subjected to the calculation of Kaplan-Meier estimators, and the comparison of the results in different groups was performed by conducting the log-rank test. We conducted subgroup analyses to further explore the therapeutic outcomes based on age, sex, race, year of diagnosis, tumor size, histology, education, and median income level among the patients in the matched group. The R software package (version 3.4.1) and SAS software (version 9.4; SAS Institute, Cary, NC) were used to perform all the calculations.

## Results

### Factors associated with the use of RFA and SBRT

A total of 6,195 nonsurgical and non-chemotherapy patients with stage IA NSCLC were identified during the 10-year period from 2004 to 2015, of whom 191 patients (3.1%) received RFA and 6,004 (96.9%) patients received SBRT as the primary treatment modality. Details about the treatment selection are presented in **Figure 1.**

The baseline characteristics of the patients are shown in** Table 1**. There were significant differences in the household income, education level, tumor size, geographical region, year of diagnosis and histology between SBRT and RFA in the unadjusted cohorts. The was no significant difference in the characteristics of the unadjusted cohorts, including the race, gender, primary labeled, Grade, Laterality, Marital status. With respect to the covariate, the two groups reached a good sound balance in propensity-score matched treatment (**Table 1**). Nonetheless, the clinical characteristics barely showed any significant difference in the adjusted cohorts.

### Factors affecting treatment selection

As demonstrated in **Table 2**, selection of the treatment method was related not only to the clinicopathological features of the patients but to their socio-economic conditions. The probability of choosing RFA as the treatment method decreased with the increase of the tumor size (>2, <3 cm, OR; 0.303; 95% CI, 0.191-0.479; >3 cm, OR; 0.153; 95% CI, 0.093-0.251) compared with the tumor size <1 cm. Patients who were admitted in hospitals in Northwest and West China and between 2004 and 2007 were more likely to accept RFA (Odds Ratio; 2.342: 95% CI, 1.595-3.438 and OR; 2.342: 95% CI, 0.255-0.504, *P*<0.0001). There was no significant difference in the household income and education level between the two groups (**Table 2**).

### Survival analysis

**Figure 2:**
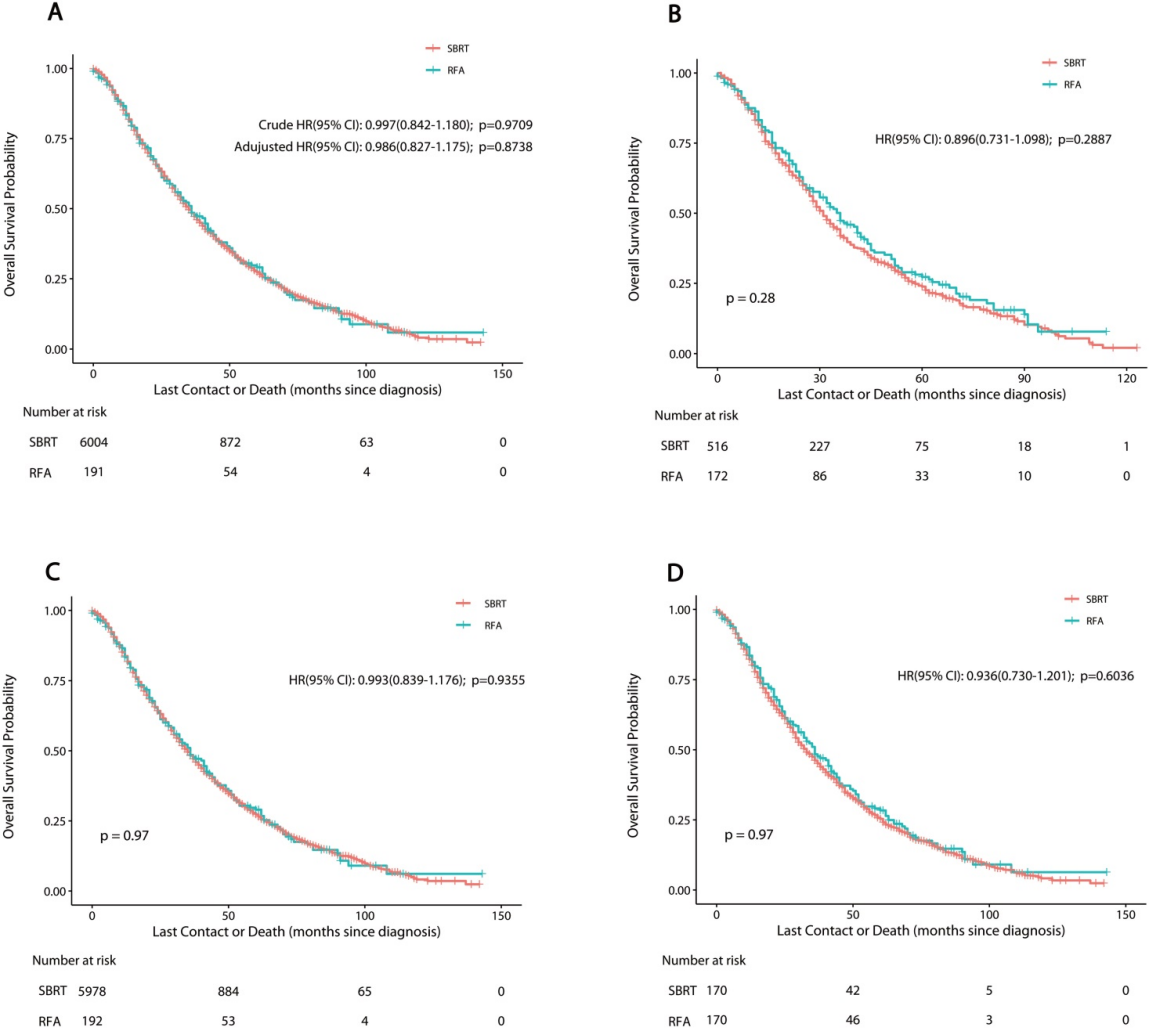
Overall survival in nonsurgically managed patients with stage IA NSCLC. A: The unmatched analysis; B: The propensity score matched analysis; C: The inverse probability of treatment weight-adjusted analysis; D: The overlap weighting analysis. Abbreviations: RFA: radiofrequency ablation; SBRT: stereotactic body radiotherapy.

The median post-treatment follow-up period was 20 months (interquartile range 9-37 months). The median OS was 36 months for RFA, and 35 months for SBRT. The one-, three- and five-year OS rate was 83.3%, 48.5% and 29.1% for RFA* vs.* 83.8%, 48.3% and 27.4% for SBRT, respectively. PSM, IPTW and Overlap Weighting analysis revealed similar results (**Table 3**). The curves of stage-specific OS are illustrated in** Figure 2.**

The results of CSS curves are shown in **Figure S1 and Table S1 (online only)**. The median time of CSS was 62 months for RFA, and 58 months for SBRT. The results of the log-rank analysis showed a barely significant difference in CSS and OS in the unadjusted cohort (*P*>0.05). The one-, three- and five-year CSS rate was 91%, 66.7% and 52.0% for RFA *vs.* 90.5%, 65.6% and 48.1% for SBRT, respectively. PSM, IPTW and Overlap Weighting analysis revealed the similar results.

**Figure 3:**
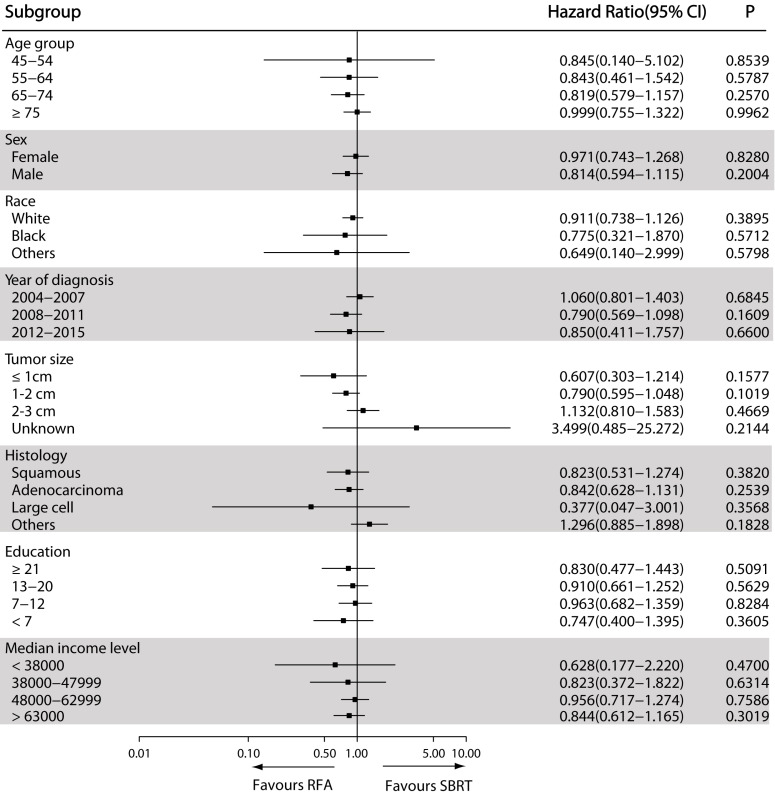
Forest plot depicting hazard ratios of RFA versus SBRT for nonsurgically managed stage IA NSCLC in the propensity score-matched population. Abbreviations: RFA: radiofrequency ablation; SBRT: stereotactic body radiotherapy.

The results of the exploratory sub-group analysis in the matched cohort showed consistent results about the impact of RFA on OS in sub-groups examined, with insignificant HR heterogeneity. The propensity-matched HRs based on different facilities, clinical and demographic features between SBRT and RFA groups are shown in **Figure 3.** Patients receiving RFA seemed to have better OS than those receiving SBRT, though the difference was not statistically significant. This specific trend was even more obvious in patients with tumors <1 cm in diameter (*P*=0.1577) (**Figure 3**).

## Discussion

In this study, we used the SEER Database to conduct a series of factor analyses on the effectiveness of RFA* vs.* SBRT in the treatment of stage IA NSCLC patients who were ineligible for surgery. The results demonstrated that RFA did not adversely affect the survival benefit in terms of OS and CSS as compared with SBRT before and after IPTW, PSM and overlap weighting method. Patients receiving RFA seemed to have better OS than those receiving SBRT, especially in patients with tumors < 1 cm in diameter.

SBRT is traditionally considered the most appropriate treatment for NSCLC patients who are ineligible for surgery or refuse to accept surgical treatment. The effectiveness of SBRT in treating stage I NSCLC has been confirmed by large numbers of previous studies 12-16. Their results showed that the tumor control rate of SBRT was comparable to that of surgery, especially in patients with inoperable early NSCLC. However, the clinical outcome of OS in patients receiving SBRT has been unsatisfactory due to the high chances of untreated lung cancer progress, underlying conditions such as chronic lung diseases, and poor general conditions of the patients. However, the tolerable dose of SBRT for risky organs, appropriateness of dose-fractionation, and other oncology-related issues remain undefined due to insufficient case samples and short periods of follow-up observation 17,18. In the cohort of RFA, a larger number of comorbidities may also be taken into account. Besides the effectiveness, the cost is another essential factor that should be taken into account in selection of relevant therapies. Generally, SBRT treatment calls for a complex plan for following treatment, assurance of high quality and complex procedures of delivery, which may result in costs beyond the support of the general healthcare system 19. There have been very few studies that have focused on the cost-effectiveness of SBRT treatment in the case of NSCLC [20.21]. Even though so far SBRT is still the primary choice in treating inoperable NSCLC patients, the application of RFA is being popularized. Some studies maintained that RFA lacked cost-effectiveness in treating inoperable NSCLC patients as compared with SBRT 22.

In our opinion, RFA has many advantages. Firstly, as an image-guided technique of thermal ablation, RFA is the most chosen treatment. PET-CT, CT, or sometimes biopsy is used to determine the recurrence 25-30. In addition, RFA has proven to have high feasibility and safety as a non-surgical NSCLC treatment. RFA is able to eradicate tumors with the thermal effect without causing much damage to nearby tissues. Ambrogi et al 25 reported that RFA could lower mortality without significantly impair the pulmonary function. There was no pneumothorax that was delayed. In a series comprising one thousand sessions of RFA 31, there was a low chance (1.6%) for pneumothorax, suggesting that RFA could be safely and effectively applied to the treatment of patients with inoperable NSCLC.

Our study showed that nonsurgical IA NSCLC patients receiving RFA seemed to have better five-year survival than those receiving SBRT, though the difference was not statistically significant (OS, HR; 0.986; 95% CI, 0.827-1.175, *P*=0.8738; CSS, HR; 0.965; 95% CI, 0.765-1.219,* P*=0.7663). The one-, three-, and five-year OS rate was 83.3%, 48.5% and 29.1% for RFA *vs.* 83.8%, 48.3% and 27.4% for SBRT respectively, showing no significant differences between the two modalities (*P*>0.05). Lam et al 10 reported that the one-, three- and five-year OS in stage IA and IB NSCLC patients was 89.3%, 52.7% and 27.1% for RFA *vs.* 85.5%, 54.3% and 31.9% for SBRT, respectively. Earlier research failed to report tumor size with RFA in NSCLC. In our study, patients receiving RFA seemed to have better OS as compared with those receiving SBRT, especially in patients with tumors <1 cm in diameter. A number of recent studies have reported promising results about cancer rates, ranging between 60% and 69%. It is clear that tumor size is an important factor in deciding the therapeutic outcome. It is generally believed that SBRT can provide better local control of large tumors, but it does not mean that SBRT can equally provide better survival. Further research should concentrate on factors that have more clinical relevance such as OS in evaluating the efficacy of various ablative treatments. We hope that the data obtained in the present study could be used as a bench-mark for further relevant research.

With the improvement of the multimodality image fusion and navigation technologies, the interventional radiology has also improved remarkably 23,24, which is very much likely to improve the effectiveness and accuracy of RFA-based treatment. However, there is no solid evidence to prove that the enhancement in interventional radiology has actually resulted in a better RFA treatment outcome in inoperable NSCLC patients. The result of analysis in our study showed no significant difference in CSS and OS between SBRT and RFA in inoperable NSCLC patients.

When it comes to the research design and the source of the research data, there are undeniably some limitations in the present study. In spite of the gradual popularization of RFA in the treatment of unresectable localized NSCLC, there has not yet been any report about the randomized controlled trials which compare the OS of patients after being treated with RFA. Even though randomized clinical trials cannot be completely replaced by observational studies, these studies still provide a good chance to fill in the gap of professional knowledge and provide answers for those questions that randomized trials are still trying to address 11. Secondary, SEER provides no data of cancer recurrence, for which the authors failed to make necessary analysis on relevant points. Besides, the entire study focused on the analysis of CCS and OS, but there are some other factors, including patient tolerance to invasive treatments, tumor location, toxicity, and cost-effectiveness, which should also be included in the discussion.

Despite the above limitations, the present study also possesses some obvious strengths. To the best of our knowledge, this is the largest cohort study to compare the therapeutic efficacy of SBRT and RFA for the treatment of inoperable NSCLC patients by using IPTW, PSM and overlap weighting methods. Our data suggest that RFA does not seem to adversely affect CSS and OS of stage IA NSCLC patients who are ineligible for surgery as compared with SBRT, which is also consistent with the result of all sub-group analyses in our exploratory investigation.

Generally speaking, our findings suggest that there's no difference of OS and CSS in patients who receive nonoperative treatment of RFA versus SBRT for stage IA NSCLC. Although our results are limited by systemic biases related to the retrospective study, especially under the condition of lacking of a randomized clinical trial, we still believe that our findings could be considered into account while local ablative therapy is the optimal treatment for localized unresectable NSCLC. Rigorous prospective randomized studies are needed to in the future to determine the role of SBRT and then optimize patient selection in this population.Our study showed no significant difference in OS and CSS between stage IA NSCLC patients receiving RFA and those receiving SBRT. Although our results may be limited by systemic biases related to the retrospective study, we still believe that our findings could be considered into account RFA is the optimal treatment for localized unresectable NSCLC, especially for those with tumors <1 cm. Rigorous prospective randomized studies are needed in future to determine the role of RFA in stage IA NSCLC patients and optimize patient selection in this population.

## Supplementary Material

Supplementary figures and tables.

## Figures and Tables

**Table 1:** Demographic and clinical characteristics of patients with lung cancer

Characteristics	Before propensity score matching			After propensity score matching			Standardized difference			
	SBRT (n=6004)	RFA (n=191)	*P*	SBRT (n=516)	RFA (n=172)	*P*	Unmatched	Matched	IPTW	Overlap weighting
Age at diagnosis, Mean (SD)	74.48 (8.89)	74.37 (9.03)	0.8200	74.50 (8.83)	74.16 (9.11)	0.6337	-0.0130	-0.0376	-0.0158	-0.0571
**Age (n,%)**			0.9621			0.7930	0.0648	0.0852	0.0675	0.1103
≤44	6 (0.10)	0 (0.00)		0 (0.00)	0 (0.00)					
45-54	110 (1.83)	3 (1.57)		5 (0.97)	3 (1.74)					
55-64	730 (12.16)	26 (13.61)		61 (11.82)	23 (13.37)					
65-74	1964 (32.71)	62 (32.46)		181 (35.08)	60 (34.88)					
≥75	3194 (53.20)	100 (52.36)		269 (52.13)	86 (50.00)					
**Race (n,%)**			0.1477			0.5141	0.1914	0.1055	0.1945	0.1800
White	5156 (85.88)	175 (91.62)		455 (88.18)	157 (91.28)					
Black	574 (9.56)	10 (5.24)		43 (8.33)	10 (5.81)					
Others	265 (4.41)	6 (3.14)		18 (3.49)	5 (2.91)					
Unknown	9 (0.15)	0 (0.00)		0 (0.00)	0 (0.00)					
Female (n,%)	3387 (56.41)	111 (58.12)	0.6403	298 (57.75)	99 (57.56)	0.9645	-0.0344	0.0039	-0.0326	-0.0299
**Year of diagnosis**			<0.0001			0.9287	0.8863	0.0337	0.8264	<0.0001
2004-2007	866 (14.42)	86 (45.03)		206 (39.92)	67 (38.95)					
2008-2011	1814 (30.21)	67 (35.08)		203 (39.34)	67 (38.95)					
2012-2015	3324 (55.36)	38 (19.90)		107 (20.74)	38 (22.09)					
**Region**			<0.0001			0.8467	0.5661	0.0798	0.5187	<0.0001
EAST	2673 (44.52)	51 (26.70)		167 (32.36)	51 (29.65)					
NORTHWEST or WEST	2312 (38.51)	125 (65.45)		298 (57.75)	106 (61.63)					
NORTH	855 (14.24)	12 (6.28)		41 (7.95)	12 (6.98)					
SOUTHWEST	164 (2.73)	3 (1.57)		10 (1.94)	3 (1.74)					
**Primary labeled**			0.4304			0.2542	0.1744	0.2010	0.1762	0.1853
Upper lobe	3715 (61.88)	109 (57.07)		322 (62.40)	98 (56.98)					
Middle lobe	270 (4.50)	12 (6.28)		17 (3.29)	11 (6.40)					
Lower	1892 (31.51)	68 (35.60)		165 (31.98)	61 (35.47)					
NOS	96 (1.60)	1 (0.52)		9 (1.74)	1 (0.58)					
Overlapping	11 (0.18)	0 (0.00)		0 (0.00)	0 (0.00)					
Main	20 (0.33)	1 (0.52)		3 (0.58)	1 (0.58)					
**Grade**			0.4802			0.6820	0.1394	0.1318	0.1332	0.0754
I	503 (8.38)	17 (8.90)		38 (7.36)	17 (9.88)					
II	994 (16.56)	23 (12.04)		78 (15.12)	22 (12.79)					
III	1212 (20.19)	41 (21.47)		102 (19.77)	39 (22.67)					
Undifferentiated	33 (0.55)	2 (1.05)		4 (0.78)	1 (0.58)					
Unknow	3262 (54.33)	108 (56.54)		294 (56.98)	93 (54.07)					
**Laterality**			0.2958			0.3280	-0.0774	-0.0866	-0.0718	-0.0721
Right	3418 (56.93)	116 (60.73)		290 (56.20)	104 (60.47)					
Left	2586 (43.07)	75 (39.27)		226 (43.80)	68 (39.53)					
Paired site	0 (0.00)	0 (0.00)		0 (0.00)	0 (0.00)					
**Histology**			0.0021			0.2597	0.2918	0.1812	0.2804	0.1874
Squamous	2017 (33.59)	41 (21.47)		147 (28.49)	37 (21.51)					
Adenocarcinoma	2897 (48.25)	101 (52.88)		242 (46.90)	92 (53.49)					
Large-cell	68 (1.13)	3 (1.57)		10 (1.94)	2 (1.16)					
Others	1022 (17.02)	46 (24.08)		117 (22.67)	41 (23.84)					
**Tumor size**			<0.0001			0.8417	0.4807	0.0798	0.4609	<0.0001
≤1 cm	272 (4.53)	29 (15.18)		73 (14.15)	23 (13.37)					
1-2 cm	2926 (48.73)	105 (54.97)		291 (56.40)	92 (53.49)					
2-3 cm	2795 (46.55)	55 (28.80)		147 (28.49)	55 (31.98)					
Unknown	11 (0.18)	2 (1.05)		5 (0.97)	2 (1.16)					
**Insurance Recode**			<0.0001			0.1772	0.5856	0.2167	0.5561	0.2046
Medicaid	627 (10.44)	6 (3.14)		41 (7.95)	6 (3.49)					
Uninsured	30 (0.50)	0 (0.00)		2 (0.39)	0 (0.00)					
Unknown	705 (11.74)	63 (32.98)		151 (29.26)	50 (29.07)					
Insured	4642 (77.32)	122 (63.87)		322 (62.40)	116 (67.44)					
**Marital status**			0.0903			0.3881	0.2424	0.1829	0.2380	0.1998
Married	2582 (43.00)	79 (41.36)		224 (43.41)	72 (41.86)					
Single	650 (10.83)	13 (6.81)		55 (10.66)	12 (6.98)					
Divorced	786 (13.09)	34 (17.80)		74 (14.34)	32 (18.60)					
Widowed	1707 (28.43)	61 (31.94)		145 (28.10)	52 (30.23)					
Unknown	270 (4.50)	4 (2.09)		18 (3.49)	4 (2.33)					
Unmarried or domestic partner	9 (0.15)	0 (0.00)		0 (0.00)	0 (0.00)					
**Education**			0.0002			0.9066	0.3288	0.0659	0.3038	0.0000
≥21	758 (12.62)	27 (14.14)		70 (13.57)	25 (14.53)					
13-20	1810 (30.15)	79 (41.36)		197 (38.18)	68 (39.53)					
7-12	2952 (49.17)	64 (33.51)		199 (38.57)	61 (35.47)					
<7	484 (8.06)	21 (10.99)		50 (9.69)	18 (10.47)					
**Median income level**			<0.0001			0.9320	0.4223	0.0587	0.3911	<0.0001
<38000	356 (5.93)	5 (2.62)		18 (3.49)	5 (2.91)					
38000-47999	1019 (16.97)	12 (6.28)		39 (7.56)	12 (6.98)					
48000-62999	2245 (37.39)	99 (51.83)		253 (49.03)	82 (47.67)					
>63000	2384 (39.71)	75 (39.27)		206 (39.92)	73 (42.44)					

Abbreviations: RFA: radiofrequency ablation; SBRT: stereotactic body radiotherapy; IPTW: inverse probability of treatment weight.

**Table 2:** Propensity Modeling of Receipt of RFA

Characteristics	OR	95% CI	*P*
**Year of diagnosis**			
2004-2007	Reference		
2008-2011	0.359	0.255 to 0.504	<.0001
2012-2015	0.111	0.075 to 0.166	<.0001
**Region**			
EAST	Reference		
NORTHWEST or WEST	2.342	1.595 to 3.438	<.0001
NORTH	0.563	0.284 to 1.117	0.1003
SOUTHWEST	0.956	0.289 to 3.164	0.9407
**Tumor size**			
≤1 cm	Reference		
≤2 cm	0.303	0.191 to 0.479	<.0001
≤3 cm	0.153	0.093 to 0.251	<.0001
Unknown	1.395	0.280 to 6.966	0.6846
**Education**			
≥21	Reference		
13-20	1.428	0.873 to 2.336	0.1553
7-12	0.738	0.425 to 1.282	0.2814
<7	1.784	0.888 to 3.585	0.1041
**Median income level**			
<38000	Reference		
38000-47999	0.736	0.249 to 2.175	0.5797
48000-62999	2.411	0.911 to 6.376	0.0762
>63000	1.586	0.569 to 4.423	0.3777

Abbreviations: RFA: radiofrequency ablation; OR: Odds Ratio.

**Table 3:** Overall Survival Rates (%) of RFA versus SBRT in patients with NSCLC

Year	Unmatched (95% CI)		Matched (95% CI)		IPTW (95% CI)		Overlap Weighting (95% CI)	
	RFA	SBRT	RFA	SBRT	RFA	SBRT	RFA	SBRT
1	83.3 (78.1-88.8)	83.8 (82.8-84.8)	83.2 (77.7-89.1)	81.5 (78.1-85.0)	83.4 (78.2-88.9)	83.8 (82.8-84.8)	83.6 (78.1-89.4)	82.2 (76.5-88.3)
2	63.5 (56.8-70.9)	64.3 (62.9-65.7)	62.9 (55.8-70.8)	61.5 (57.2-66.0)	63.7 (57.0-71.1)	64.3 (62.9-65.7)	63.7 (56.6-71.6)	62.1 (54.9-70.2)
3	48.5 (41.6-56.6)	48.3 (46.8-49.9)	47.3 (40.0-55.9)	41.9 (37.6-46.7)	48.5 (41.6-56.6)	48.3 (46.8-49.8)	48.4 (41.1-57.0)	46.2 (38.8-55.0)
4	38.0 (31.2-46.2)	36.5 (35.0-38.2)	36.0 (28.9-44.7)	32.5 (28.4-37.2)	37.7 (31.0-45.9)	36.5 (34.9-38.1)	37.2 (30.1-46.0)	34.3 (27.3-43.1)
5	29.1 (22.8-37.1)	27.4 (25.8-29.1)	27.3 (20.7-35.8)	23.8 (20.0-28.4)	29.0 (22.7-37.0)	27.3 (25.7-29.0)	28.4 (21.8-37.0)	25.4 (18.9-34.0)

Abbreviations: RFA: radiofrequency ablation; SBRT: stereotactic body radiotherapy; IPTW: inverse probability of treatment weight.

## Abbreviations

NSCLC: non-small cell lung cancer
RFA: radiofrequency ablation
SBRT: stereotactic body radiotherapy
OS: overall survival
CSS: cancer-specific survival
IPTW: inverse probability of treatment weight
PSM: propensity score-matched
SEER: Surveillance, Epidemiology, and End Results registry
OR: odds ratio
HR: hazard ratio
CMH: Cochran-Mantel-Haenszel

## References

[1] Siegel RL, Miller KD, Jemal A (2019). Cancer statistics, 2019. CA Cancer J Clin.

[2] Gorenstein LA1, Sonett JR (2011). The surgical management of stage I and stage II lung cancer. Surg Oncol Clin N Am.

[3] Hirsh J, Guyatt G, Albers GW (2008). American College of Chest Physicians. Executive summary: American College of Chest Physicians Evidence-Based Clinical Practice Guidelines (8th Edition). Chest.

[4] San José S, Arnaiz MD, Lucas A (2006). Radiation therapy alone in elderly with early stage non-small cell lung cancer. Lung Cancer.

[5] Chen M, Hayman JA, Ten Haken RK (2006). Long-term results of high-dose conformal radiotherapy for patients with medically inoperable T1-3N0 non-small-cell lung cancer: is low incidence of regional failure due to incidental nodal irradiation?. Int J Radiat Oncol Biol Phys.

[6] Dupuy DE (2013). Treatment of medically inoperable non-small-cell lung cancer with stereotactic body radiation therapy versus image-guided tumor ablation: can interventional radiology compete?. J Vasc Interv Radiol.

[7] Ahmed M, Brace CL, Lee FT (2011). Principles of and advances in percutaneous ablation. Radiology.

[8] Liu B, Liu L, Hu M (2015). Percutaneous radiofrequency ablation for medically inoperable patients with clinical stage I non-small cell lung cancer. Thorac Cancer.

[9] Bi N, Shedden K, Zheng X (2016). Comparison of the effectiveness of radiofrequency ablation with stereotactic body radiation therapy in inop- erable stage I non-small cell lung cancer: a systemic review and pooled analysis. Int J Radiat Oncol Biol Phys.

[10] Lam A, Yoshida EJ, Bui K (2018). A National Cancer Database Analysis of Radiofrequency Ablation versus Stereotactic Body Radiotherapy in Early-Stage Non-Small Cell Lung Cancer. J Vasc Interv Radiol.

[11] Booth CM, Tannock IF (2014). Randomised controlled trials and population-based observational re- search: Partners in the evolution of medical evidence. Br J Cancer.

[12] Shioyama Y, Onishi H, Takayama K (2018). Japanese Radiological Society Multi-Institutional SBRT Study Group (JRS-SBRTSG). Clinical Outcomes of Stereotactic Body Radiotherapy for Patients With Stage I Small-Cell Lung Cancer: Analysis of a Subset of the Japanese Radiological Society Multi-Institutional SBRT Study Group Database. Technol Cancer Res Treat.

[13] Frakulli R, Salvi F, Balestrini D (2017). Radiological differential diagnosis between fibrosis and recurrence after stereotactic body radiation therapy (SBRT) in early stage non-small cell lung cancer (NSCLC). Transl Lung Cancer Res.

[14] Miyakawa A, Shibamoto Y, Baba F (2017). Stereotactic body radiotherapy for stage I non-small-cell lung cancer using higher doses for larger tumors: results of the second study. Radiat Oncol.

[15] Temming S, Kocher M, Stoelben E (2018). Risk-adapted robotic stereotactic body radiation therapy for inoperable early-stage non-small-cell lung cancer. Strahlenther Onkol.

[16] Kimura T, Nagata Y, Harada H (2017). Phase I study of stereotactic body radiation therapy for centrally located stage IA non-small cell lung cancer (JROSG10-1). Int J Clin Oncol.

[17] Guckenberger M (2014). Stereotactic body radiotherapy for stage I NSCLC: the challenge of evidence-based medicine. J Thorac Oncol.

[18] Onishi H, Araki T (2013). Stereotactic body radiation therapy for stage I non-small-cell lung cancer: a historical overview of clinical studies. J Clin Oncol.

[19] Hirsch JA, Schaefer PW, Romero JM (2014). Comparative effectiveness research. AJNR Am J Neuroradiol.

[20] Paix A, Noel G, Falcoz PE (2018). Cost-effectiveness analysis of stereotactic body radiotherapy and surgery for medically operable early stage non-small-cell lung cancer. Radiother Oncol.

[21] Lester-Coll NH, Sher DJ (2017). Cost-Effectiveness of Stereotactic Radiosurgery and Stereotactic Body Radiation Therapy: a Critical Review. Curr Oncol Rep.

[22] Pollom EL, Lee K, Durkee BY (2017). Cost effectiveness of stereotactic body radiation therapy versus radiofrequency ablation for hepatocellular carcinoma: A Markov modeling study. Radiology.

[23] Ahmed M, Brace CL, Lee FT Jr (2011). Princi- ples of and advances in percutaneous ablation. Ra- diology.

[24] Wood BJ, Kruecker J, Abi-Jaoudeh N (2010). Navigation systems for ablation. J Vasc Interv Radiol.

[25] Ambrogi MC, Fanucchi O, Cioni R (2011). Long-term results of radiofrequency ablation treatment of stage I non- small cell lung cancer: a prospective intention-to-treat study. J Thorac Oncol.

[26] Beland MD, Wasser EJ, Mayo-Smith WW Primary non-small cell lung cancer: review of frequency, location, and time of recurrence after radiofrequency ablation. Radiology.2010:254:301-307.

[27] Fernando HC, De HA, Landreneau RJ (2015). Radiofrequency ablation for the treatment of non-small cell lung cancer in marginal surgical candidates. J Thorac Cardiovasc Surg.

[28] Hiraki T, Gobara H, Iishi T (2007). Percutaneous radiofrequency ablation for clinical stage I non-small cell lung cancer: results in 20 nonsurgical candidates. J Thorac Cardiovasc Surg.

[29] Lanuti M, Sharma A, Digumarthy SR (2009). Radiofrequency ablation for treatment of medically inoperable stage I non-small cell lung cancer. J Thorac Cardiovasc Surg.

[30] Pennathur A, Luketich JD, Abbas G (2007). Radiofrequency ablation for the treatment of stage I non-small cell lung cancer in high-risk patients. J Thorac Cardiovasc Surg.

[31] Kashima M, Yamakado K, Takaki H (2011). Complications after 1000 lung radiofrequency ablation sessions in 420 patients: a single center's experiences. AJR Am J Roentgenol.

[32] Palussière J, Chomy F, Savina M (2018). Radiofrequency ablation of stage IA non-small cell lung cancer in patients ineligible for surgery: results of a prospective multicenter phase II trial. J Cardiothorac Surg.

[33] Simon CJ, Dupuy DE, DiPetrillo TA (2009). Pulmonary radiofrequency ablation: long-term safety and efficacy in 153 patients. Radiology.

[34] Hiraki T, Gobara H, Iishi T (2007). Percutaneous radiofrequency ablation for clinical stage I non-small cell lung cancer: results in 20 nonsurgical candidates. J Thorac Cardiovasc Surg.

[35] Lanuti M, Sharma A, Digumarthy SR (2009). Radiofrequency ablation for treatment of medically inoperable stage I non-small cell lung cancer. J Thorac Cardiovasc Surg.

[36] Pennathur A, Luketich JD, Ghulam A (2007). Radiofrequency ablation for the treatment of Stage I non-small cell lung cancer in high-risk patients. J Thorac Cardiovasc Surg.

