# Supplementary material for: Radiofrequency ablation vs. stereotactic body radiotherapy for stage IA non-small cell lung cancer in nonsurgical patients
Source: J Cancer. 2021 Mar 19;12(10):3057–66. doi: 10.7150/jca.51413 (PMC8040894; doi:10.7150/jca.51413)
Supplement: Supplementary file 1 — Supplementary figures and tables. [file jcav12p3057s1.pdf]

Figure S1

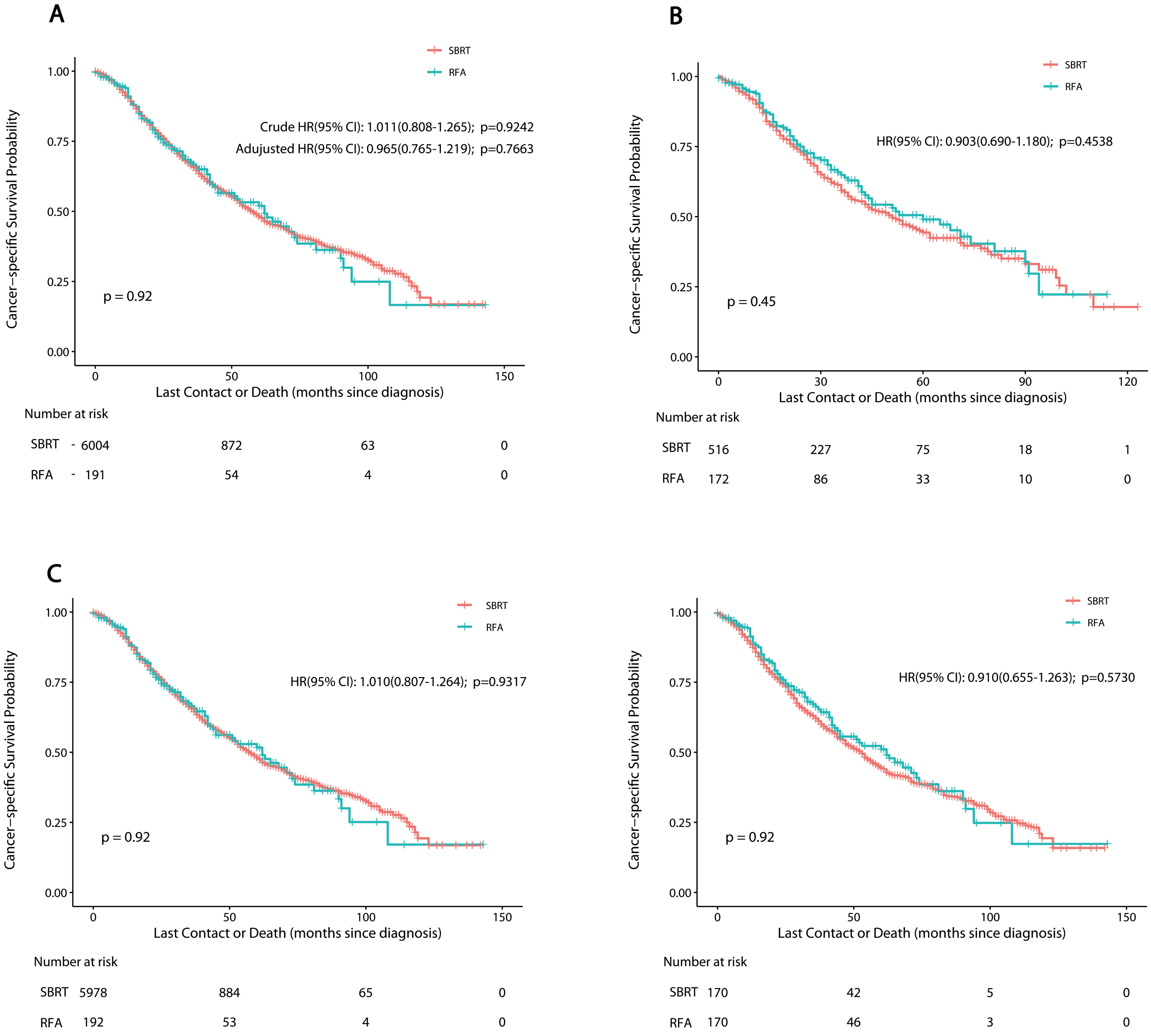
A
SBRT
RFA
Crude HR(95% CI): 1.011(0.808-1.265); p=0.9242
Adujusted HR(95% CI): 0.965(0.765-1.219); p=0.7663
p = 0.92
Cancer−specific Survival Probability
1.00
0.75
0.50
0.25
0.00
0
50
100
150
Last Contact or Death (months since diagnosis)
Number at risk
SBRT - 6004 872 63 0
RFA - 191 54 4 0
B
SBRT
RFA
HR(95% CI): 0.903(0.690-1.180); p=0.4538
p = 0.45
Cancer−specific Survival Probability
0
30
60
90
120
Last Contact or Death (months since diagnosis)
Number at risk
SBRT 516 227 75 18 1
RFA 172 86 33 10 0
C
SBRT
RFA
HR(95% CI): 1.010(0.807-1.264); p=0.9317
p = 0.92
Cancer−specific Survival Probability
Last Contact or Death (months since diagnosis)
Number at risk
SBRT 5978 884 65 0
RFA 192 53 4 0
SBRT
RFA
HR(95% CI): 0.910(0.655-1.263); p=0.5730
p = 0.92
Cancer−specific Survival Probability
Last Contact or Death (months since diagnosis)
Number at risk
SBRT 170 42 5 0
RFA 170 46 3 0

sTable 1. Cancer-specific Survival Rates (%) of RFA versus SBRT in Patients with NSCLC

Year	Unmatched (95% CI)		Matched (95% CI)		IPTW (95% CI)		Overlap Weighting (95% CI)	
	RFA	SBRT	RFA	SBRT	RFA	SBRT	RFA	SBRT
1	91.0 (86.8-95.3)	90.5 (89.7-91.3)	90.5 (86.1-95.2)	88.8 (86.0-91.6)	91.1 (87.0-95.4)	90.4 (89.6-91.2)	91.2 (86.9-95.8)	88.6 (83.8-93.7)
2	75.7 (69.4-82.6)	76.9 (75.6-78.2)	75.0 (68.2-82.4)	73.0 (68.9-77.3)	75.9 (69.6-82.7)	76.8 (75.6-78.1)	75.8 (69.1-83.1)	74.3 (67.5-81.9)
3	66.7 (59.6-74.7)	65.6 (64.0-67.2)	64.9 (57.2-73.5)	59.3 (54.6-64.4)	66.5 (59.4-74.5)	65.5 (63.9-67.1)	66.2 (58.6-74.8)	62.0 (54.1-71.0)
4	56.5 (48.7-65.7)	56.4 (54.5-58.2)	54.3 (45.9-64.1)	51.7 (46.7-57.2)	56.2 (48.3-65.3)	56.2 (54.4-58.1)	55.6 (47.2-65.4)	52.4 (44.0-62.4)
5	52.0 (43.8-61.7)	48.1 (46.1-50.3)	49.0 (40.3-59.6)	44.3 (39.0-50.4)	51.7 (43.5-61.4)	48.0 (46.0-50.2)	51.0 (42.4-61.5)	44.4 (35.5-55.4)

Abbreviations: RFA, radiofrequency ablation; SBRT, stereotactic body radiotherapy; IPTW, inverse probability of treatment weight.
